# Salivary Fractalkine Differentiating Periodontitis from Periodontally Healthy Subjects

**DOI:** 10.1155/2024/3007148

**Published:** 2024-04-01

**Authors:** Omer Mohammed Harbood, Raghad Fadhil Abbas

**Affiliations:** Department of Periodontology, College of Dentistry, University of Baghdad, Baghdad, Iraq

## Abstract

**Objective:**

The chemokine “Fractalkine” (CX3CL1) and its corresponding receptor (CX3CR1), chemokine belonging to the CX3C family, have an essential role in developing several systemic inflammatory disorders. Accordingly, the proliferation, adhesion, and migration of inflammatory cells are all affected by it. In light of this, the present study attempts to address the following questions: (1) Is the salivary level of fractalkine and its receptor associated with periodontitis patients with different severities? (2) Is it possible to distinguish periodontitis from periodontally healthy subjects?

**Methods:**

This study included 30 individuals who had been considered controls, having healthy periodontium, and 90 patients with varying stages of periodontitis. The patients were equally divided into three groups: those with Stage I, Stage II, and Stage III. After each subject's saliva was collected, periodontal markers including bleeding on probing (BOP), probing pocket depth (PPD), and clinical attachment loss (CAL) were recorded. Enzyme-linked immunosorbent assays (ELISA) were used to detect the protein levels of salivary CX3CL1 and CX3CR1.

**Results:**

In comparison to the control group, patients with periodontitis had statistically increased salivary concentrations of CX3CL1 and CX3CR1 (*P*  < 0.001). Additionally, all clinical periodontal indicators (BOP, PPD, and CAL) had a strong association with salivary CX3CL1 and CX3CR1 levels. Furthermore, by using the ROC (receiver operating characteristic), both biomarkers showed a good ability to differentiate periodontitis from periodontally healthy subjects, and an excellent ability to distinguish Stage I and Stage III periodontitis from periodontally healthy subjects. The AUC for salivary CX3L1 and its receptors, CX3R, was 0.93 and 0.8, respectively, to distinguish Stage I from patients with good periodontal health. In contrast, the biomarkers' AUC for separating individuals with Stage III periodontitis from those in healthy periodontal conditions was 1.

**Conclusion:**

Fractalkine and its receptor are linked to periodontitis and may distinguish between periodontitis and healthy periodontal tissues, suggesting its role as a possible part of periodontal disease pathogenesis.

## 1. Introduction

Complex etiopathogenesis is thought to underlie damaging inflammatory diseases such as periodontitis [1]. Hence, periodontal disease is believed to arise from a range of interactions between the biofilm found in the pathogenic periodontal pockets and the immune-inflammatory responses of the host [2]. An inflammation-specific attraction of immune cells to the affected area occurs in the context of response to periodontal inflammation, which is mediated by different cytokine patterns [3]. In addition to proinflammatory cytokines, chemokines, a set of chemotactic molecules, also contribute to the initiation of this reaction. This response is regulated by the expression of adhesion molecules on various cells, such as endothelial cells and keratinocytes [4]. Accordingly, the inflammatory cells are activated and drawn into inflamed tissues [5].

One of the most important chemokines is Fractalkine (CX3CL1) which comes in two different forms: soluble glycoprotein and membrane-bound glycoprotein. The main kinds of cells which exhibit CX3CL1 are osteoblasts, fibroblasts, and endothelial cells. CX3CL1 binds to CX3CR1, which is expressed on cytotoxic effector lymphocytes, monocytes/macrophages, and osteoclasts, performing several functions such as chemotaxis, enhancement of other chemokines, boosting macrophage survival, and enhancing adhesive and migratory functions of leukocytes [6, 7].

Saliva has traditionally been considered a reliable indicator of the general well-being of the body due to its inclusion of serum elements that are assessed in standard blood tests for disease detection and health monitoring. As a diagnostic fluid, saliva offers numerous advantages over serum for disease diagnosis. The collection is noninvasive, thereby making it simpler, safer, and more cost-effective than blood withdrawal. Saliva collection can be done without the assistance of trained persons, unlike blood collection [1].

CX3CL1 interaction with its receptors seems to mediate pathophysiological processes during inflammatory events [6]. Diabetes mellitus and rheumatoid arthritis are systemic diseases that are associated with an increased amount of CX3CL1 and CX3CR1, providing good evidence for their role in the chronic inflammatory process and pathogenesis of bone resorption. Indeed, these diseases are closely related to periodontitis. The investigation of CX3CL1 and CX3CR1 levels in the blood and gingival crevicular fluid (GCF) of periodontitis patients has been exclusively examined by Balci et al. [8] in 2021 within the context of clinical research. However, the current study evaluating their concentrations in saliva in periodontitis patients with different severities has been conducted. Therefore, this study seeks to assess the correlation between Fractalkine (CX3CL1) and the receptor for it (CX3CR1), as well as the different stages of periodontitis, to differentiate individuals with periodontitis from those who are periodontally healthy.

## 2. Methods and Materials

### 2.1. Design of Study

In the Department of Periodontics' teaching clinics, observational case-control research was conducted at the University of Baghdad/College of Dentistry from December 2022 to April 2023. Furthermore, the Ethics Commission at the (University of Baghdad/College of Dentistry) granted its official approval to the study (project no 733622 on 1-12-2023) after thoroughly reviewing the study's design and gathering detailed information. Upon being provided with an elaborate explanation of the study's objectives and procedures, all participants were requested to affix their signature on a consent document.

### 2.2. Grouping and Sample Size Determination

Using the first samples taken from each group in a 1 : 1 : 1 : 1 allocation ratio, a pilot study was carried out. Enzyme-linked immunosorbent assay (ELISA) techniques were used for protein analysis of salivary samples. Consequently, the necessary sample size was calculated using the mean and standard deviation data obtained from this pilot study according to the equation mentioned by Sharma et al. [9] in 2020 for the case-control study. While the sample size for the healthy control group was 30, the projected sample size for the periodontitis group was 80, which was rounded up to 90 to prevent attrition bias. Accordingly, each periodontitis group consisted of around 30 patients. The grouping and case definition of the controls and cases were as follows:

Periodontal health including the following criteria to identify the control subjects as having healthy periodontium: bleeding on probing; BOP < 10%, probing pocket depth; PPD ≤ 3 mm, and intact periodontium [10].

Stage I periodontitis: 1–2 mm of clinical attachment loss (CAL) and radiographic bone loss not exceeding 15%.

Stage II periodontitis: involves patients when the CAL is 3–4 mm or in the coronal third of the root where the percentage of bone loss is between 15% and 33% of root length.

Stage III periodontitis: including patients with Stage III periodontitis, which affects the middle third of the root due to extensive bone loss, as the bone loss involves 34%–66% of root length. The examples for the current study were represented by the last three categories.

### 2.3. Inclusion Criteria

Periodontitis groups were defined following by Tonetti et al. [10] in 2018. Buccal or oral CAL is found in two teeth with pocketing >3 mm, while interdental CAL can be seen in two nonadjacent teeth. All periodontitis patients should demonstrate a generalized form (30% of teeth were affected) of Grade B or C (calculated by dividing the percentage of bone loss by the age of the patients if the result is more than 1 the grade will be C while if the result lies between 0.5 and 1 the grade is considered as B), and an unstable state (PPD 5 or PPD 4 mm with BOP). Consequently, the current study comprised participants who did not have any systemic diseases (save for the case criteria given above), were nonsmokers, had more than 20 teeth, and were willing to participate. Moreover, each patient enrolled in the study had their body mass index (BMI) calculated, with the weight subdivided by the “height square” (kg/m^2^) to exclude overweight subjects.

### 2.4. Exclusion Criteria

Subjects with dental implants, deep carious lesions, systemic or inflammatory illnesses including Crohn's disease, liver or renal dysfunction, a history of organ transplantation or cancer treatment, any disease or disorder affecting the cardiovascular system or the blood vessels supplying the kidneys, and alcoholics were excluded from the study. People who were undergoing active orthodontic or periodontal therapy, had periapical inflammation, had recently taken antibiotics or immunosuppressants, were taking long-term hormone contraceptives or similar medications, had salivary glands diseases, were pregnant or nursing mothers, or had any oral lesions unrelated to periodontitis were also disqualified.

### 2.5. Periodontal Parameters and Clinical Examination

Each participant's complete periodontal charting, including BOP (+/−), PPD, and CAL, was documented by a calibrated examiner. Except for wisdom teeth, we assessed six particular sites per tooth (mesio-buccal, mid-buccal, disto-buccal, mesio-lingual, mid-lingual, and disto-lingual) using a periodontal probe (UNC-15) to collect clinical periodontal data. Measurements of PPD, CAL, and BOP were performed during the calibration sessions. The measurement between the researcher and specialist in periodontics was performed after 2 hr to avoid false positive results if it is conducted immediately, the calibration will be repeated until the results of agreement are more than 75%.

### 2.6. Collection of Salivary Samples

It was instructed to participants to fast for 1 or 2 hr before the collection of saliva. To remove any particles or impurities before collecting saliva, the participants were asked to thoroughly rinse their mouths with tap water 30 min before beginning the collection process. Next, the participants were instructed to maintain an upright posture while tilting their heads forward. Saliva was collected from the participants' lower lips and deposited into a plastic cup. Each individual yielded around 3 mL of saliva. Subsequently, the entire amount of saliva obtained was drawn out from a disposable cup utilizing a micropipette to remove 500 *μ*L of saliva, which was then transferred into a plastic tube. Subjecting the obtained samples to centrifugation at a speed of 1,000 rpm for 15 min facilitated the elimination of cellular waste from the salivary supernatants. A micropipette was used to aspirate 500 mL of the clear salivary supernatants into a plastic Eppendorf tube. The tube already contained 50 mL of a protease inhibitor enzyme solution. After labeling, the Eppendorf tubes were frozen at −20°C until they were analyzed.

### 2.7. Enzyme-Linked Immunosorbent Assays (ELISA)

The samples were thawed and left for a few minutes to reach room temperature. Commercial ELISA assays kits were used to determine the amounts of salivary Fractalkine protein (CX3CL1) and its receptor CX3CR1 protein (fine-test). The process was carried out according to each kit's manufacturer's instructions. The detection threshold for the biomarker CX3L1 is 1–156 ng/mL, while the threshold for CX3R1 is 125–8,000 pg/mL. An optical density measurement at a wavelength of 450 nm was obtained using a microtiter plate reader called HumaReader HS, manufactured by the HUMAN Society for Biochemica and Diagnostica mbH. Using a linear regression technique customized for each biomarker, all optical density measurements were exported to spreadsheets and converted to concentrations

## 3. Statistical Analyses

The software GraphPad Prism 9.5.1 was utilized for data description, analysis, and presentation. The ANOVA test was used to evaluate information from many groups, and the findings showed that they had a distribution that was normal according to the Shapiro–Wilk test. If the results were found to be statistically significant, we conducted additional comparisons between different groups using Dunnett's T3 test. Pearson correlation was used to determine the relationship between the biomarkers and clinical periodontal measures. The diagnostic accuracy of both biomarkers was assessed using the area under the curve (AUC) and receiver operating characteristic (ROC), and discrimination values and ranges as good, excellent, or outstanding according to Hosmer and Lemeshow [11] in 2000. A *p* value was considered statistically significant if it was below 0.05.

## 4. Results

**Figure 1:**
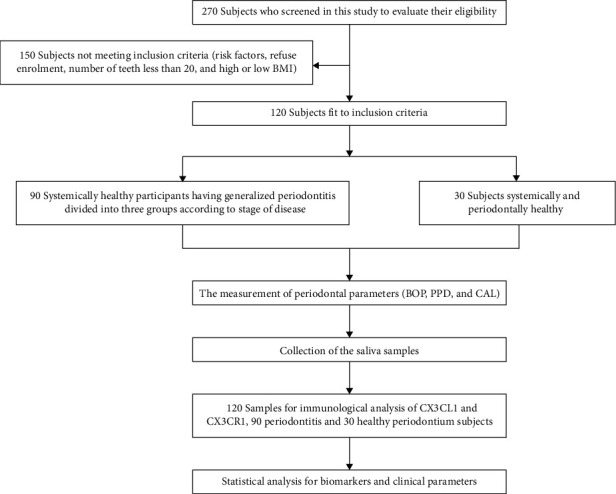
Flow chart for study design.

The current study involved 120 individuals in all, eligible to be included in the current work divided into four groups (Figure 1).

**Figure 2:**
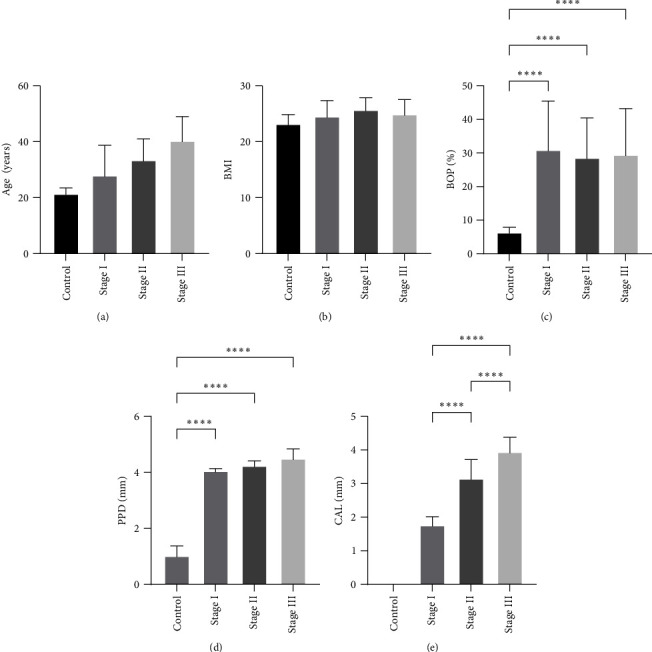
Distribution and comparison of demographic and clinical parameters. Age and BMI were shown in graphs (a) and (b), whereas the comparison of clinical periodontal parameters among groups was shown in graphs (c–e). BOP%: bleeding on probing percentage; PPD: probing pocket depth; CAL: clinical attachment loss.  ^*∗∗∗∗*^ significant at *p*-value < 0.001; presentation done with bar chat with standard deviation from the mean values involved in the study.

The frequency distribution of the participants by sex, mean participant age, mean BMI, and clinical periodontal features in each group are displayed in Table 1 and Figure 2.

**Figure 3:**
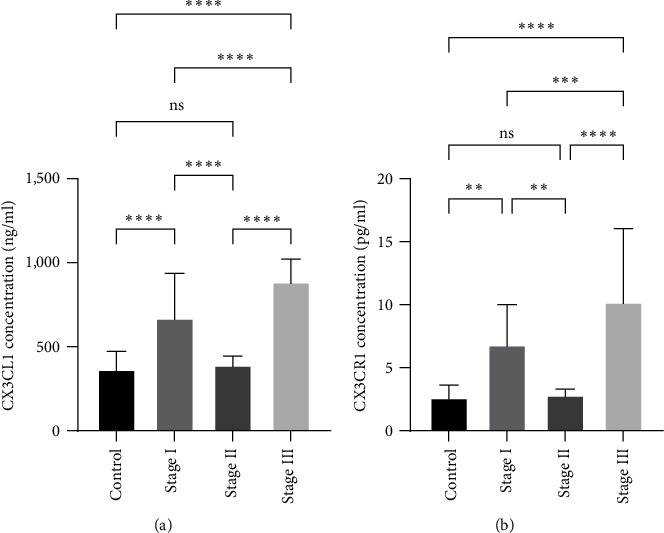
Comparisons of salivary biomarkers among groups: (a) salivary concentration of CX3CL1 among groups and (b) salivary CX3CR1 concentration among groups.  ^*∗∗*^ significant at *p*-value < 0.05;  ^*∗∗∗*^ significant at *p*-Value < 0.01;  ^*∗∗∗∗*^ significant at *p*-value < 0.001; ns: nonsignificant at *p*-Value ≥ 0.05; presentation done with bar chat with standard deviation from mean values of salivary biomarkers involved in the study.

Statistical analyses for both salivary biomarkers (CX3CL1 and CX3CR1) are illustrated in Table 2. In contrast to the control group, those with higher levels of periodontitis at various stages showed statistically significant results. Additionally, significant alteration was detected between different stages of periodontitis. Still, the difference for both biomarkers was statistically significant between the control group with Stage I and Stage III, while a nonsignificant difference was found with Stage II, as illustrated by Figures 3(a) and 3(b).

Considering the analysis of both biomarkers between different grades of periodontitis the current study illustrates higher concentration in grade C periodontitis than grade B yet the result was nonsignificant between them as shown in Table 3.

Concerning the correlation of salivary biomarkers with each other and with clinical periodontal parameters, the result has shown a significant association between both biomarkers with PPD and CAL in the periodontitis group, and a significant association was detected between both biomarkers. However, a nonsignificant association was found between both biomarkers and BOP, as presented in Table 4.

**Figure 4:**
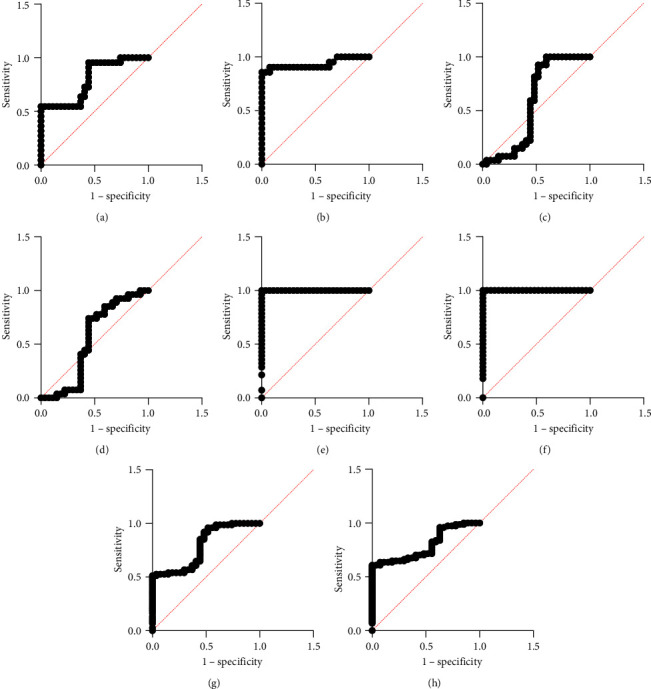
Receiver operating characteristics (ROC) curves of both biomarkers (CX3CL1 and CX3CR1): (a) control versus Stage I periodontitis for CX3CL1, (b) control versus Stage I periodontitis for CX3CR1, (c) control versus Stage II periodontitis for CX3CL1, (d) control versus Stage II periodontitis for CX3CR1. (e) control versus Stage III periodontitis for CX3CL1, (f) control versus Stage III periodontitis for CX3CR1, (g) control versus periodontitis for CX3CL1, and (h) control versus periodontitis for CX3CR1.

Finally, the result of diagnostic accuracy of both biomarkers to discriminate periodontal health from periodontitis with different severities as illustrated in Table 5. As AUC for salivary CX3L1 and its receptors CX3R was 0.794 and 0.796, respectively, pointing out how they help distinguish between periodontitis and periodontal health. Furthermore, the above-mentioned biomarkers showed high diagnostic accuracy in differentiating Stage I and Stage III from periodontal health, yet they failed to distinguish Stage II from the healthy controls.  Figure 4(a)–4(d) below demonstrates that when assessing periodontal health Stages I and III, the two biomarkers were more sensitive and specific than when contrasting Stage II.

## 5. Discussion

For the first time, this study compared healthy controls to patients with varying degrees of periodontitis and measured the amounts of protein (CX3CL1) and the receptor (CX3CR1) in their saliva. Both biomarkers were significantly higher in the periodontitis group than in the healthy control group. The strong positive association observed between the biomarkers and clinical periodontal parameters (PPD and CAL) in patients with periodontitis provides significant support for these findings. Indicating that this chemokine could play a role in the development of periodontal diseases and localized inflammatory reactions. In addition, both biomarkers emerged as a good candidate for distinguishing periodontal health from a different stage of periodontitis especially Stage I as it is considered a borderline between gingivitis and periodontitis, making the diagnosis by clinical evaluation sometimes quite difficult.

According to the findings of the study, CX3CL1 could be involved in the inflammatory degradation of periodontal tissues. This finding suggests that salivary CX3CL1 levels may have increased due to local tissue inflammation [6]. Chemokines collectively mediate the activation of inflammatory cascade reactions in tissue damage, playing a crucial role during inflammation. CX3CL1 is a unique chemotactic factor that can exist in soluble and membrane-bound forms among more than 200 chemokines. CX3CR1, its only receptor, belongs to the G protein-coupled receptor superfamily [7]. By interacting with many inflammatory signaling pathways, including Toll-like receptors, NF-*κ*B, Wnt/*β*-catenin, and others, the CX3CL1 and CX3CR1 may both influence a variety of inflammatory processes [12, 13]. Thus, by acting as both a chemotactic factor and an adhesion molecule for osteoclast precursors expressing CX3CR1, CX3CL1 facilitates osteoclast recruitment and development. It should be noted that osteoblasts express CX3CL1, whereas osteoclast precursors express CX3CR1. The differentiation of osteoclasts from osteoblasts is mediated by CX3CL1, which is found only on osteoblasts [14].

According to several studies, CX3CL1 has a significant impact on the inflammation associated with rheumatoid arthritis. Higher soluble and membrane-bound levels of CX3CL1 and CX3CR1 have been found in the serum of rheumatoid arthritis patients, suggesting that CX3CL1 is the main factor that drives inflammation through the recruitment of monocytes [15, 16]. Rheumatoid arthritis and periodontitis are both chronic diseases that share some comparable pathological processes, such as chemokine profiles characterized by elevated amounts of proinflammatory cytokines [17]. Thus, a proinflammatory/anti-inflammatory cytokine imbalance akin to that observed in individuals with rheumatoid arthritis may be the cause of the elevated amounts of Fractalkine and its receptor in the saliva of periodontitis patients. The concentration of both biomarkers was higher in grade C in comparison with grade B, yet the result was nonsignificantly altered between both grades of periodontitis. This could be ascribed to different stages included in each grade group masking the result of biomarkers as it was significantly altered among different stages of periodontitis.

Furthermore, nonsignificant alteration between Stage II and healthy control was found in the current work. Indeed, numerous researchers have documented that multiple routes can stimulate various chemokines, which have a regulatory function in periodontal disease and bone remodeling in both normal and abnormal situations [13, 18]. Accordingly, Periodontitis is a multifaceted, chronic inflammatory disease characterized by nonlinear progress. It is caused by multiple causes that act simultaneously and interact with one another. Several factors contribute to the immunological fitness of an individual. The host exists in a symbiotic relationship with the oral microbiome to maintain homeostasis [19–21]. explaining the fluctuation of some chemokines levels during disease progression in favor of elevation of others.

It was unclear which pathway is responsible for the increased salivary CX3CL1 that leads to induction of bone resorption as the current study was an observational case-control study. To elucidate these mechanisms of periodontitis, additional research at the molecular level, such as experimental or cell line investigations, is required. This is considered as a major limitation for the current study, furthermore omitting significant risk factors like smoking as well as the effect of local and systemic disease might affect the validity of the current result so further study is required to confirm the present study [22, 23].

Yet, there is no study investigating the concentrations of CX3CL1 and CX3R1 in saliva about periodontitis with different severities as well as different progression rates, nonetheless, similar results were obtained for both biomarkers investigated in (GCF) and the serum was obtained from patients with periodontitis [2, 24]. Accordingly, the severity of periodontal disease may thus affect salivary CX3CL1 and its receptors. Also, the significance of Fractalkine and its receptor as inflammatory mediators associated with periodontal disease has been underlined by the results. The significantly elevated levels of CX3CL1 and its receptor in the group with periodontitis, compared to the control group with healthy periodontal conditions, provide more evidence supporting the involvement of CX3CL1 in the development of periodontitis. Nevertheless, Further investigation is required to elucidate the precise function of fractalkine and its associated components in all stages of periodontitis, to determine the specific impact of fractalkine on the advancement of periodontal disease.

## 6. Conclusion

The presence of salivary Fractalkine and its receptor may play a role in the development of periodontitis and could be used to distinguish between Stages I and III of the disease, as well as between healthy and diseased periodontal tissues. However, the concentration of these biomarkers is not significantly affected by the rate at which periodontitis progresses.

## Figures and Tables

**Table 1:** Demographic and clinical periodontal parameters in healthy controls and patients with Stage I, Stage II, and Stage III periodontitis.

	Control		Stage I		Stage II		Stage III		
No.	30		30		30		30		
	Mean	±SD	Mean	±SD	Mean	±SD	Mean	±SD	*p*-Value
Age (years)	21.500	2.028	27.955	10.750	34.393	8.829	40.357	8.608	0.067
BMI (kg/cm^2^)	23.061	1.798	24.532	2.796	25.750	2.555	24.925	2.606	0.091
BOP (%)	6.350	1.493	30.950	14.525	28.418	11.634	31.418	12.056	0.000
PPD (mm)	2.30	1.020	4.050	0.086	4.243	0.162	4.496	0.342	0.000
CAL (mm)	0.000	0.000	1.759	0.252	3.129	0.556	3.943	0.438	0.000
Gender (*N* %)									
M	15	53.57	14	63.64	21	75.00	23	82.14	0.105
F	13	46.43	8	36.36	7	25.00	5	17.86	

All results in Table 1 were obtained from the ANOVA test except for sex which was obtained from the *chi*-square test. BMI: body mass index; BOP: bleeding on probing; PPD: probing pocket depth; CAL: clinical attachment loss; No.: number of participants; M: male; F: female; SD: standard deviations. Level of significance as not significant *p*  > 0.05, significant at *p*  < 0.05. Value except the sex was in mean ± standard deviations.

**Table 2:** Statistical analysis of the concentration of salivary biomarkers among all groups.

		Mean	SD	Minimum	Maximum	*p*-Value
CX3CL1 (ng/mL)	Control	363.779	104.932	180.930	559.114	0.000
	Stage I	670.440	266.821	279.888	981.535	
	Stage II	389.303	52.618	315.846	547.628	
	Stage III	939.853	242.325	628.970	1,539.470	

CX3CR (pg/mL)	Control	2.634	0.958	0.573	3.775	0.000
	Stage I	7.205	3.607	2.200	15.936	
	Stage II	2.842	0.468	1.234	3.455	
	Stage III	10.413	6.128	3.940	21.210	

ANOVA test; CX3CL1: Fractalkine ligand; CX3CR: Fractalkine receptor; SD: standard deviations; Level of significance: significant at *p*  < 0.001.

**Table 3:** Statistical analysis of the concentration of salivary biomarkers between grade B and grade C.

	Grades								
	B				C				
	Min	Max	Mean	SD	Min	Max	Mean	SD	*p*-Value
CX3CL1	325.9	1,200.900	650.468	54.359	279.888	1,539.470	673.667	45.146	0.760
CX3CR1	1.234	17.659	5.909	0.887	1.869	21.210	7.206	0.762	0.309

Analysis done using *t*-test; CX3CL1: Fractalkine ligand; CX3CR1: Fractalkine receptor; SD: standard deviations; Min: minimum; Max: maximum; Level of significance: significant at *p*  < 0.05.

**Table 4:** Correlation between salivary biomarkers and clinical periodontal parameters.

		BOP (%)		PPD		CAL		CX3CL1	
		*r*	*p*	*r*	*p*	*r*	*p*	*r*	*p*
Periodontitis	CX3CL1	0.030	0.798	0.414	0.000	0.326	0.004	—	—
	CX3CR1	−0.080	0.488	0.388	0.000	0.283	0.012	0.761	0.000

Pearson correlation; CX3CL1: Fractalkine ligand; CX3CR1: Fractalkine receptors; BOP%: bleeding on probing percentage; PPD: probing pocket depth; CAL: clinical attachment loss; Level of significance: significant at *p* < 0.01.

**Table 5:** Diagnostic accuracy of biomarkers among groups.

	Variable	AUC	*p*-Value	Optimal cutoff value	Sensitivity (%)	Specificity (%)
Control/stage I	CX3CL1	0.8	0.000	258.64	100	85.7
	CX3CR1	0.938	0.000	0.739	100	92.9

Control/stage II	CX3CL1	0.583	0.287	370.297	60.7	46.4
	CX3CR1	0.482	0.819	2.879	46.4	53.6

Control/stage III	CX3CL1	1	0.000	192.695	100	96.4
	CX3CR1	1	0.000	0.627	100	96.4

Control/periodontitis	CX3CL1	0.794	0.000	390.18	78.2	42.9
	CX3CR1	0.796	0.000	2.879	78.2	53.6

CX3CL1: Fractalkine ligand; CX3CR1: Fractalkine receptors; AUC: area under the curve.

## Data Availability

Data availability was available on request.
